# Functions of the CSB Protein at Topoisomerase 2 Inhibitors-Induced DNA Lesions

**DOI:** 10.3389/fcell.2021.727836

**Published:** 2021-10-22

**Authors:** Franciele Faccio Busatto, Sofiane Y. Mersaoui, Yilun Sun, Yves Pommier, Jean-Yves Masson, Jenifer Saffi

**Affiliations:** ^1^Laboratory of Genetic Toxicology, Federal University of Health Sciences of Porto Alegre (UFCSPA), Porto Alegre, Brazil; ^2^Post-Graduation Program in Molecular and Cell Biology (PPGBCM), Federal University of Rio Grande do Sul (UFRGS), Porto Alegre, Brazil; ^3^Oncology Division, CHU de Québec-Université Laval, Quebec City, QC, Canada; ^4^Laval University Cancer Research Center, Quebec City, QC, Canada; ^5^Developmental Therapeutics Branch, Laboratory of Molecular Pharmacology, Center for Cancer Research, National Cancer Institute, National Institutes of Health, Bethesda, MD, United States

**Keywords:** Topoisomerase 2 inhibitors, Nucleotide Excision Repair (NER), R-loops, DNA repair, CSB, Topoisomerase 2

## Abstract

Topoisomerase 2 (TOP2) inhibitors are drugs widely used in the treatment of different types of cancer. Processing of their induced-lesions create double-strand breaks (DSBs) in the DNA, which is the main toxic mechanism of topoisomerase inhibitors to kill cancer cells. It was established that the Nucleotide Excision Repair pathway respond to TOP2-induced lesions, mainly through the Cockayne Syndrome B (CSB) protein. In this paper, we further define the mechanism and type of lesions induced by TOP2 inhibitors when CSB is abrogated. In the absence of TOP2, but not during pharmacological inhibition, an increase in R-Loops was detected. We also observed that CSB knockdown provokes the accumulation of DSBs induced by TOP2 inhibitors. Consistent with a functional interplay, interaction between CSB and TOP2 occurred after TOP2 inhibition. This was corroborated with *in vitro* DNA cleavage assays where CSB stimulated the activity of TOP2. Altogether, our results show that TOP2 is stimulated by the CSB protein and prevents the accumulation of R-loops/DSBs linked to genomic instability.

## Introduction

Topoisomerase are essential enzymes required for transcription, replication, and chromatin remodeling. Topoisomerases TOP1 and TOP2 mediate the cleavage, respectively, of single or double stranded DNA for relaxing generated DNA supercoiling, untangle catenanes, and condense chromosomes, avoiding DNA over winding. Topoisomerases are particularly vulnerable to topoisomerase poison (topoisomerase inhibitors) during their cleavage reaction. These drugs block the re-ligation step of the enzyme-induced DNA break through the formation of the drug-DNA-topoisomerase complexes, which is referred to as the cleavage complex (TOPcc). The cytotoxic activity of TOP1 inhibitors such as camptothecin is related to the interference of trapped TOP1cc with DNA replication and transcription. Processing of these complexes creates double-strand breaks (DSBs) in the DNA, which is their main toxic mechanism to kill cancer cells (Pommier, 2013; Pommier et al., 2016; Marinello et al., 2018). Similarly, Topoisomerase 2 (TOP2) inhibitors such as Doxorubicin (DOX) and Mitoxantrone (MXT) are drugs widely used in the treatment of different types of cancer, such as breast, prostate, lung, bladder, testis, leukemia, lymphomas, and osteosarcomas.

We have previously reported that NER pathway deficiency reenforces TOP2 inhibitors suggesting a role of the (NER) pathway in processing the TOP2cc intermediate (Saffi et al., 2010; Rocha et al., 2016a, b). Thus, deficiency in Cockayne syndrome B (CSB), a protein from TC-NER (Transcription Coupled – NER), accumulates more Top2ccs in response to MXT than cells deficient in XPC, a protein from GG-NER (Global Genome – NER) (Rocha et al., 2016a). These results strongly indicate an involvement of the NER pathway, or at least of CSB, in processing of these complexes and, maybe, mediating the generation of DSBs.

The CSB protein, also known as ERCC6, is a multifunctional protein belonging to the SWI/SNF2 superfamily that completes other non-canonical functions besides the classical functions NER pathway, including DSBs repair (Batenburg et al., 2015). Batenburg et al. (2017) have shown that CSB is involved in the pathway choice to repair DSBs, once it removes histones from the damaged area in the DNA, allowing HR proteins to access it. It was also shown that CSB could imply in DSBs signaling when they occur in active-transcribed genes, once these are important regions in the genome (Wei et al., 2015, 2016; Teng et al., 2018). CSB also seems to be involved in resolving R-loops (Sollier et al., 2014), which are DNA:RNA hybrids that can occur physiologically during different processes, including transcription and replication (Chédin, 2016; Bhatia et al., 2017). Persistent R-loops forming in the head-on direction can block DNA replication and, if unresolved, can cause DNA breaks and genomic instability (Aguilera and Gómez-González, 2017; Hamperl et al., 2017). CSB is also involved in recognizing such hybrids at active-transcribed regions, promoting mRNAs release for their use as a template by HR factors (Teng et al., 2018).

It has also been shown that R-loops are powerful and reversible topology sink that cells may use to relieve superhelical stress during transcription (Stolz et al., 2019). Coordinated action of Top1 and Top2 counteract the accumulation of torsional stress at replication forks, thus preventing the diffusion of topological changes along large chromosomal regions (Bermejo et al., 2007). Hence cells treated with Camptothecin increases topological stress which accumulate R-loops and result into more genome instability (Sollier et al., 2014; Manzo et al., 2018). We hypothesized that CSB recognizes Top2cc mediated R-loops in response to TOP2 inhibitors. Such R-loops accumulation might be a consequence of RNA Polymerase (RNA Pol) arrest causing by the complexes TOP2ccs. Therefore, this study aimed to understand the role of CSB in response to TOP2 inhibitors and the relation with DSB repair pathways.

## Materials and Methods

### Cell Culture, siRNAs, Plasmids and Transfection

All mammalian cells were cultured at 37°C with 5% CO_2_. U2OS human osteosarcoma cells (ATCC) was cultured in McCoy‘s medium and HEK-293T cells (ATCC) and U2OS-TRE reporter cells were cultured in Dulbecco’s modified Eagle’s medium, all of them containing 10% fetal bovine serum (FBS). All cells were transfected with plasmid DNAs using Lipofectamine 2000 and siRNA oligonucleotides using Lipofectamine RNAiMAX (Invitrogen) according to the manufacturer’s instructions. All siRNAs were purchased from Dharmacon as a SMARTpool and 50 nM siRNA was used for transfection. pTREX-FLAG-TOP2A and pTREX-FLAG-TOP2B were provided from Dr. YP lab (NIH) and 1 μg DNA was used to overexpress TOP2A and TOP2B in HEK-293T cells. pBroad3 TA-KR and pBroad3 tetR-KR were provided by Dr. Li Lan lab (MGH) and 1 μg DNA was used for damage at RNA transcription (DART) assay.

### Reagents and Antibodies

Doxorubicin (DOX), Mitoxantrone (MXT) and Etoposide (ETO) were purchased from Sigma Aldrich. Antibodies anti-CSB (ab96089), anti-topoisomerase 2 alpha (ab12318) and anti-topoisomerase 2 beta (ab72334) were purchased from Abcam. Monoclonal anti-FLAG M2 antibody and monoclonal anti-vinculin antibody (V9131), used as loading control in western blot analysis, were purchased from Sigma Aldrich. Anti-phospho-histone H2A.X (Ser139) (clone JBW301) was purchased from Merck Millipore and 53BP1 antibody was purchased from Novus Biologicals (NB100-304). For DRIP and DART experiments, S9.6 antibody was purified from the hybridoma purchased from the American Type Culture Collection (ATCC, Manassas, VA, United States), by Dr. Masson. ANTI-FLAG^®^ M2 Affinity Gel (Sigma Aldrich) and protein-A/G agarose beads (Pierce^TM^ Protein A/G UltraLink^TM^ Resin – Thermo Fisher Scientific) were used for CSB and TOP2 Immunoprecipitation (IP) experiments and R-Loops IP at DRIP-qPCR assay. To confirm XPC and XPA knockdown through qPCR 2 pair of primers were used for each gene as follows: 5′ TTGTCGTGGAGAAGCGGTCTAC/3′ CTTCTCCAAGCCTCACCACTCT and 5′ GACAAGCAGGA GAAGGCAAC/3′ GGTTCGGAATCCTCATCAGA for XPC; 5′ GAAGTCCGACAGGAAAACCGAG/3′ GATGAACAATCG TCTCCCTTTTCC and 5′ GCAGCCCCAAAGATAATTGA/3′ TGGCAAATCAAAGTGGTTCA for XPA. Primers used for DRIP-qPCR analysis are presented in Supplementary Table 1.

### Damage at RNA Transcription Assay

U2OS-TRE cells were first transfected with siRNAs (siERCC6, siTOP2A, and siTOP2B) and 24 h after siRNA transfection the same cells were transfected with plasmids expressing KillerRed (KR) (pBroad3 TA-KR and pBroad3 tetR-KR). 36–48 h after plasmids transfection, cells were exposed for 25 min to a 15W Sylvania cool white fluorescent lamp for ROS-induced damage through light-induced KR activation and let for 1 h to recover before fixation to start the S9.6 Immunofluorescence (IF) protocol. Cells were rinsed with PBS and fixed in 4% PFA for 15 min at room temperature. They were washed three times with PBS, permeabilized by 0.2% Triton X-100 in PBS for 10 min, and then washed three times with PBS. After that, cells were incubated in buffer (10 mM Tris-HCl, 2 mM EDTA, pH = 9) and steamed on a 95°C heating block for 20 min to expose the antigen. The dish was cooled down, washed three times with PBS and cells were blocked using 5% BSA in 0.1% PBST for 30 min at room temperature. The first and secondary antibodies were diluted in the same blocking buffer (anti-S9.6 1:500 and anti-mouse Alexa-Fluor 488 1:1,000). Primary antibody was incubated for 2 h at room temperature, then cells were washed three times with PBS and incubated for 1 h with the secondary antibody, following three more washes with PBS and incubation with DAPI 1 mg/mL. Images were taken using Volocity (Quorum Technologies) and S9.6 intensity in the KillerRed foci area was quantified using the same software.

### DRIP-qPCR

DRIP assays were performed as described by Mersaoui et al. (2018). Briefly, nucleic acids were extracted from U2OS cells by SDS/proteinase K treatment at 37°C overnight followed by phenol-chloroform extraction using MaXtract^TM^ High Density (100 × 15mL from QIAGEN) and ethanol precipitation at room temperature. The harvested nucleic acids were digested for 24 h at 37°C using a restriction enzyme cocktail (50 units/100 μg nucleic acids, each of *Bsr*GI, *Eco*RI, *Hin*dIII, *Ssp*I, and *Xba*I) in the New England Biolabs CutSmart buffer with 2 mM Spermidine and 1X BSA. Digested DNAs were cleaned up by phenol-chloroform extraction using MaXtract^TM^ High Density (200 × 2mL from QIAGEN) followed by treatment or not with RNase H (20 units/100 μg nucleic acids) overnight at 37°C in the New England Biolabs RNase H buffer. DNA:RNA hybrids from 4 μg digested nucleic acids, treated or not with RNase H, were immunoprecipitated using 10 μg of S9.6 antibody and 50 μl of protein-A/G agarose beads (Pierce^TM^ Protein A/G UltraLink^TM^ Resin – Thermo Fisher Scientific) at 4°C for overnight and 2 h, respectively, in IP buffer (10 mM NaPO_4_, 140 mM NaCl, 0.05% Triton X-100). Beads were then washed three times with IP buffer for 10 min at room temperature and nucleic acids were eluted with elution buffer (50 mM Tris-HCl, pH 8.0, 10 mM EDTA, 0.5% SDS and 70 μg of protease K) at 55°C for 1 h. Immunoprecipitated DNA were then cleaned up by a phenol-chloroform extraction followed by ethanol precipitation at −20°C for 1 h. Quantitative PCR was performed at the indicated regions using the primers listed in Supplementary Table 1. Enrichment of DNA:RNA hybrids was calculated as percentage of input.

### Immunofluorescence

For γH2AX and 53BP1 immunostaining U2OS cells were at first transfected with siERCC6, siXPC, and siXPA. 24 h after siRNA transfections cells were treated with 0.025 μg/ml DOX and 0.0125 μg/ml MXT for additional 24 h, and right after that the IF protocol was performed. Briefly cells were rinsed three times with PBS 1X, fixed using PFA 2% for 30 min, rinsed three times with PBS 1X and permeabilized with PBS-Triton X-100 0.3% for 30 min, followed by three washes with PBS 1X. Before incubation with antibodies, cells were blocked for 30 min using a blocking buffer (10% goat serum; 0.5% NP40, 0.5% saponin in PBS 1X). Primary and secondary antibodies were diluted in blocking buffer (anti-γH2AX 1:10,000, anti-53BP1 1:1,000, Alexa-fluor 488 goat anti-rabbit 1:1000 and Alexa-fluor 568 goat anti-mouse 1:1,000). Incubation with primary antibodies was done for 1 h 30 min and with secondary antibodies for 1 h. The slides were prepared using ProLong^TM^ Gold Antifade Mountant with DAPI (Thermo Fisher Scientific) to stain the nucleus. Three independent experiments were performed, and the images were taken using Volocity software (Quorum Technologies). A hundread cells per condition were analyzed for foci number using Cell Profiler software (Broad Institute). For S9.6 immunostaining the details are described in the DART assay method.

### Cell Lysis and Immunoprecipitation

For co-immunoprecipitation experiments of endogenous proteins, U2OS cells were treated with DOX or MXT for 24 h and right after lysed with a lysis buffer containing 50 mM Tris-HCL pH 7.5, 150 mM NaCl, 0.5% NP40 and a cocktail of protease inhibitors and phosphatase inhibitors. After a brief sonication (5 cycles 30 s on/off) followed by high speed centrifugation, the supernatant was precleared and protein quantification was done using Bradford method (Bradford, 1976). 1–2 mg protein lysate was separated for IP, and 0.6 μL of benzonase was added. The IP was performed incubating at first the lysate with anti-CSB and anti-IgG for the IgG control, according to the antibody manufacturer instructions, for 1 to 2 h at 4°C in rotation. After that, this lysate that was previously incubated with the antibody was then incubated for 1 h at 4°C in rotation with approximately 40 μL of beads. After IP, beads were washed with the lysis buffer (without protease and phosphatase inhibitors), and eluted. Samples were applied in a SDS-PAGE gel and blotted against anti-TOP2 or anti-CSB to check the interaction between the endogenous proteins.

For overexpression TOP2 immunoprecipitations, HEK-293T cells overexpressing FLAG tagged TOP2A and TOP2B were treated with DOX, MXT, and ETO for 2 h. After lysis and quantification following the same procedure described for endogenous proteins IP, the FLAG-tagged proteins IP was performed using 50 μL of beads ANTI-FLAG^®^ M2 Affinity Gel (Sigma Aldrich), and the lysate containing the beads was incubated for 3 h at 4°C in rotation. After IP, beads were washed with washing buffer containing 50 mM Tris-HCl pH 7.5, 250 mM NaCl, 0.5% NP40, and eluted. Samples were applied in a SDS-PAGE gel and blotted against anti-FLAG or anti-CSB to check the interaction between proteins.

### *In vitro* Topoisomerase 2 Cleavage Assay

Cockayne Syndrome B protein purification was performed by the GST-His protein purification method as described by Maity et al. (2013). Human TOP2α and TOP2β were purified from yeast strains JEL1 top1Δ transformed with 12-URA-B 6 × His-hTOP2α and 12-URA-C 6 × His-hTOP2β, respectively. Induction of TOP2 by galactose as described previously (Dong et al., 2000). Yeast cells were lysed in equilibration buffer [300 mM KCl, 10 mM imidazole, 20 mM Tris HCl pH 7.7, 10% glycerol, and protease inhibitor cocktail (Sigma-Aldrich, catalog no. P8215)] by glass bead homogenization. Lysates were incubated with Ni-NTA resin and washed using wash buffer #1 (300 mM KCL, 30 mM imidazole, 20 mM Tris HCl pH 7.7, 10% glycerol, and protease inhibitors) then wash buffer #2 (150 mM KCl, 30 mM imidazole, 20 mM Tris HCl pH 7.7, 10% glycerol, and protease inhibitor cocktail). TOP2α and β were eluted on a Poly-Prep chromatography column (Bio-Rad, catalog no. 7311550) with elution buffer (150 mM KCl, 300 mM imidazole, 20 mM Tris HCl pH 7.7, 10% glycerol, and protease inhibitors). The peak protein fractions were dialyzed in dialysis buffer (750 mM KCl, 50 mM Tris HCl pH 7.7, 20% glycerol, 0.1 mM EDTA, and 0.5 mM DTT) and His tag was removed using Tobacco Etch Virus (TEV) protease.

Topoisomerase plasmid cleavage assay was carried out as described previously (Nitiss et al., 2012). In brief, 5 nM pBR322 supercoiled plasmid DNA and 100 nM recombinant TOP2α or TOP2β were incubated in 20 μL TOP2 reaction buffer containing 20 mM Tris-HCl, pH 7.5, 10 mM MgCl_2_, 150 mM KCL, 1 mM ATP, 1 mM EDTA, 1 mM DTT, and 30 μg/mL acetylated BSA (TOP2) in the presence of 50 μM etoposide and indicated concentrations of CSB 37°C for 30 min. The reactions were terminated by adding 2 μL 10% SDS, 0.75 μL of 500 mM EDTA, pH 8.0, and 2 μL 0.8 mg/mL proteinase K and further incubated for 2 h at 30°C. DNA samples were electrophoresed in 0.8% agarose gels containing 0.5 μg/mL ethidium bromide.

### Statistical Analysis

Statistical analysis was performed with GraphPad Prism software, version 6.0 (Cherwell Scientific, Oxford, United Kingdom). Results are the mean of at least three independent experiments with error bars showing S.E.M. Statistical analysis was performed using one-way ANOVA followed by Tukey’s multiple comparison test and two-tailed Student’s *t*-test (Mann-Whitney test *U*-test). An alpha level of 0.05 was used to determine significance in all statistical analysis.

## Results

### Topoisomerase 2 Knockdown, but Not Inhibition, Increases R-Loops

**FIGURE 1:**
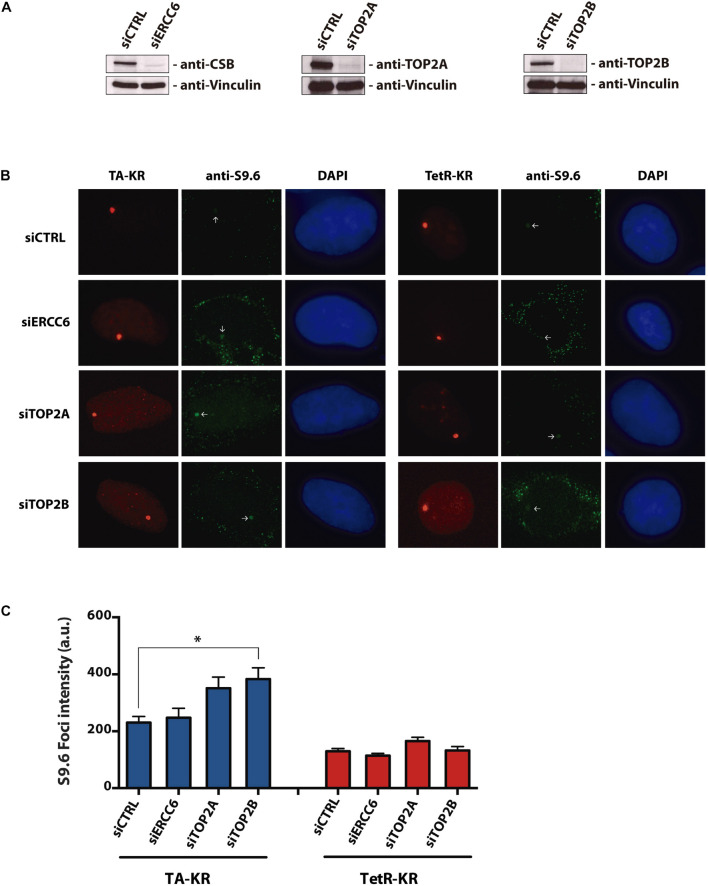
Depletion of TOP2A and TOP2B increases R-loops at transcribed regions with local ROS-induced DNA damage. **(A)** Western blotting of U2OS-TRE cells with ERCC6, TOP2A, and TOP2B knockdowns. **(B)** Representative images of S9.6 staining in siCTRL, siERCC6, siTOP2A and siTOP2B knockdown at transcription on (TA-KR) or off (tetR-KR) genomic loci in U2OS TRE cells. **(C)** Quantification of the S9.6 foci intensity in the indicated conditions. Bars represent mean of S9.6 foci intensity quantification ± SEM from three independent experiments. The statistical analysis was performed by two-tailed Student’s *t*-test (Mann-Whitney test *U*-test). 100 cells per condition were analyzed at each independent experiment.

Previous reports show an increase in R-loops formation after TOP1 inhibition by Camptothecin (Sollier et al., 2014; Marinello et al., 2016; Manzo et al., 2018) and also concerning CSB role in resolving these DNA:RNA hybrids (Sollier et al., 2014), we wanted to evaluate the R-loops accumulation in the context of CSB and TOP2 knockdowns. For that we performed the DNA DART assay in siCTRL, siERCC6 (CSB), siTOP2A, and siTOP2B cells. This system can measure a DNA:RNA hybrid accumulation at a particular locus (Lan et al., 2014; Teng et al., 2018). The light-inducible chromophore-modified KillerRed (KR) is fused with either transcription activator (TA) or repressor (tetR). KR generates reactive oxygen species (ROS) through the excited chromophore and induces DNA damage and transcriptional activation at the genome-integrated tet response element (TRE) locus in U2OS TRE cells. Elevated R-loop at the TRE locus over background is visualized by immunofluorescence using the S9.6 antibody (Lan et al., 2014; Teng et al., 2018). TOP2B knockdown led to a significant increase in R-loops specifically at the TA-KR marked locus, while the level of R-loops was similar to the control at the tetR-KR locus (Figure 1). Although it was not statistically different, we also saw an increase in TOP2A knockdown in relation to the control in TA-KR. These findings further confirm the accumulation of R-loops in the absence of TOP2A and TOP2B using an independent assay i.e., DART, in the presence or not of damage.

**FIGURE 2:**
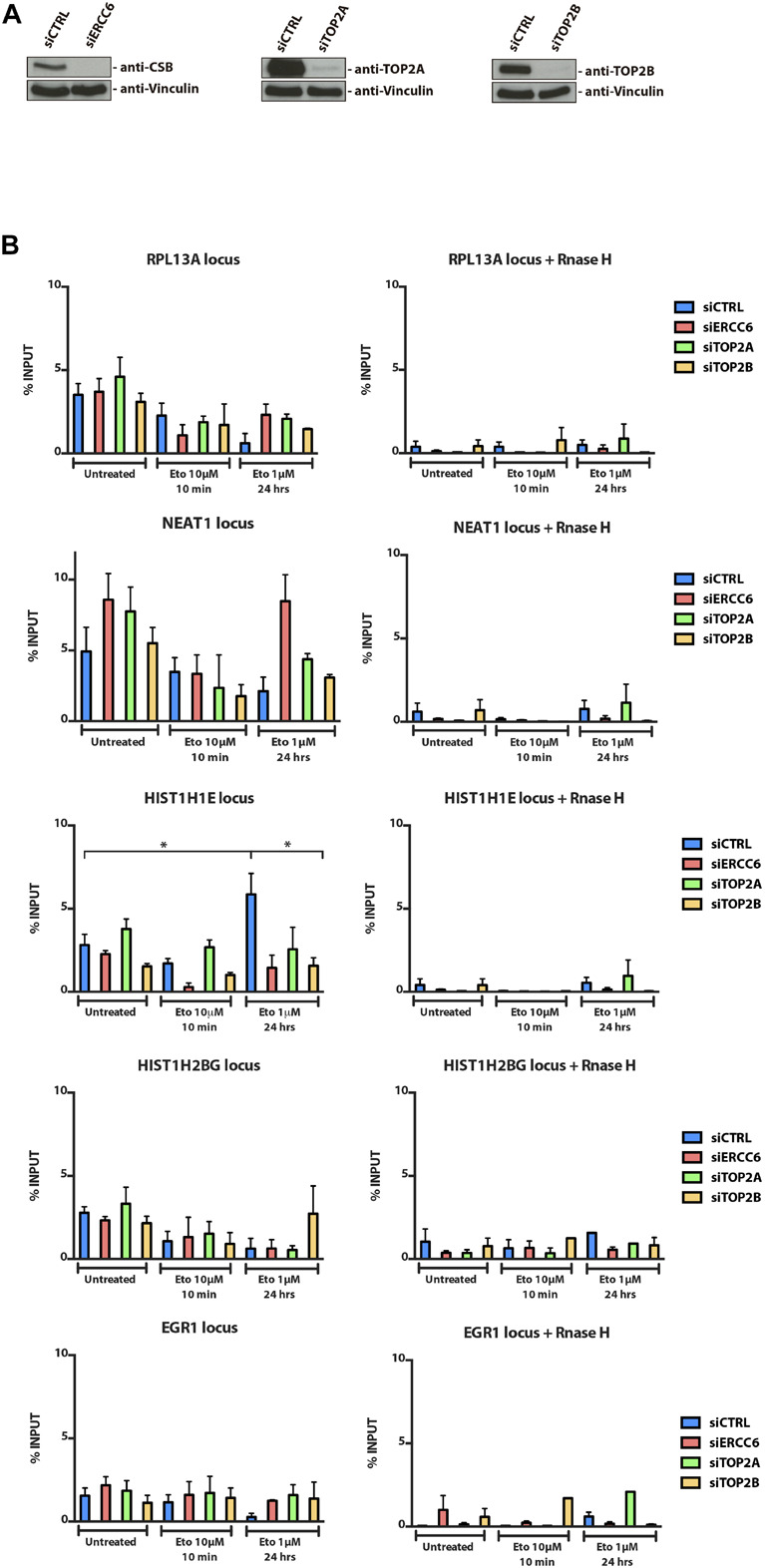
R-loops immunoprecipitation through DRIP-qPCR. **(A)** U2OS cellswere transfected with siCTRL, siERCC6, siTOP2A, and siTOP2B and **(B)** treated with ETO for 10 min and 24 h. After treatments, cells were subjected to DRIP-qPCR analysis. The average ± SEM. from three independent experiments is shown. Statistical analysis were performed using one-way ANOVA with Tukey’s multiple comparison test; *p* < 0.05 was considered as significative.

We also chose to investigate the occurrence of the same pattern after TOP2 pharmacological inhibition, which would induce different lesions from the ROS induced lesions. To assess R-loops levels in U2OS cells treated with a TOP2 inhibitor, we immunoprecipitated the DNA:RNA hybrids performing a DRIP-qPCR assay, which is a specific method to detect R-loops at different loci known to accumulate these structures. Surprisingly, in 4 out of 5 loci analyzed we did not find a significant increase in R-loops after TOP2 inhibition with ETO (Figures 2A,B) and MXT (data not shown) at any siRNA condition. Furthermore, even though we saw a significant R-loops increase in siCTRL cells treated with ETO for 24 h at HIST1H1E loci, this was not observed for other genes that were analyzed. In fact, in general, for all siRNAs tested R-loops levels were either the same or lower after ETO treatments. However, in 4 out of 5 locus there is a slight increase in siTOP2A condition in untreated cells, but when treated with ETO for short (10 min) or long exposure (24 h) we could not find a pattern.

### siERCC6 and siXPC Present Different 53BP1 and γH2AX Foci Pattern

**FIGURE 3:**
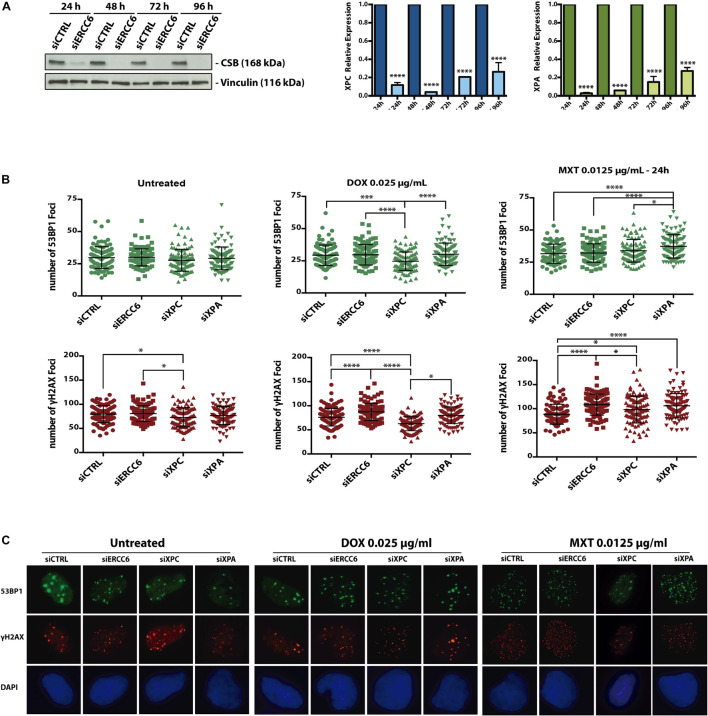
Cockayne Syndrome B depletion induces more 53BP1 foci formation upon TOP2 inhibition. U2OS cells were transfected with siCTRL, siERCC6, siXPC, and siXPA **(A)** and treated with DOX and MXT for 24 h. **(B)** represents 53BP1 and γH2AX foci quantification and **(C)** shows representative images. Graphs represents the average ± SEM from three independent experiments. Statistical analysis were performed using one-way ANOVA with Tukey‘s multiple comparison test; *p* < 0.05 was considered as significative. 100 cells per condition were analyzed at each independent experiment.

In order to determine if TOP2 inhibitor induces DSBs we assessed γH2AX and 53BP1 foci formation in U2OS cells. Then we investigated the influence NER genes in this response, after siRNA knockdown for ERCC6, XPC and XPA (Figure 3A). After 24 h treatments with DOX and MXT, we could see differences in the foci formation among the knockdowns (Figures 3B,C). After DOX treatments siXPC cells present less 53BP1 and γH2AX foci compared to siCTRL, siXPA, and specially siERCC6. The same pattern is observed in γH2AX foci at untreated and MXT conditions, when siXPC cells present less foci than siERCC6 and siCTRL. This can indicate that the absence of XPC does not affect the signaling to repair DSBs, once the evaluation was done 24 h after the treatments and by this time we could think the DSBs generated by these drugs are already resolved. On the other hand, the absence of CSB, evaluated by siERCC6, seems to increase γH2AX foci, but not 53BP1, in relation to siCTRL with both TOP2 inhibitors. This can indicate that there are more DSBs at these conditions or the absence of CSB delays the resolution of this DSBs, maintaining the phosphorylation of H2AX even after 24 h later.

### Cockayne Syndrome B Interacts With TOP2A and TOP2B

**FIGURE 4:**
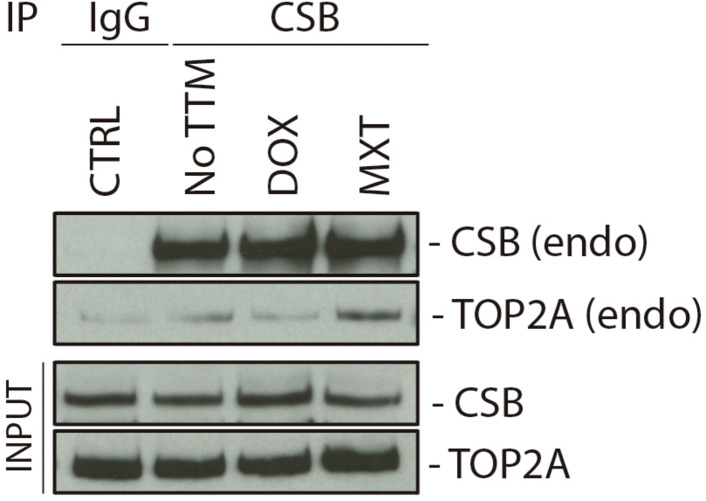
Cockayne Syndrome B physically interacts with TOP2A. U2OS cells were treated for 24 h with DOX or MXT and a CSB IP was performed after the treatments. CTRL cells did not perform CSB IP, but IgG instead. NO TTM: cells were CSB-IP, but no treatment was applied with the TOP2 inhibitors.

Considering that ERCC6 knockdown increases DSBs levels upon TOP2 inhibition (Figure 3B), and CSB depleted cells accumulate more Top2ccs than XPC-deficient cells in response to MXT we hypothesized a direct interaction between CSB and TOP2 before or after TOP2 inhibition. This interaction could be necessary to process the Top2ccs-complexes. To test this, we performed a Co-Immunoprecipitation (Co-IP) of the endogenous CSB using U2OS cells, before or after 24 h treatment with TOP2 inhibitors (DOX or MXT). Western blotting with Anti-TOP2A, revealed a slight interaction between TOP2A and CSB in normal condition (Figure 4). Interestingly, the TOP2A-CSB interaction is increased after MXT treatments.

We also evaluated this protein interaction in HEK 293T cells overexpressing both isoforms, TOP2A or TOP2B, FLAG-tagged and treated with three different TOP2 inhibitors, DOX, MXT, and ETO for 2 h. When endogenous CSB was analyzed in FLAG IP samples through western blot, we found a slight interaction between TOP2A and CSB in untreated condition that was more pronounced in cells treated with DOX and MXT, but less intense in ETO treatment (Supplementary Figure 1A). However, for TOP2B we observed a very slight interaction in untreated and more pronounced in MXT treated cells (Supplementary Figure 1B). Different from TOP2A, the same interaction was not observed in DOX and ETO treatments when TOP2B was immunoprecipitated.

**FIGURE 5:**
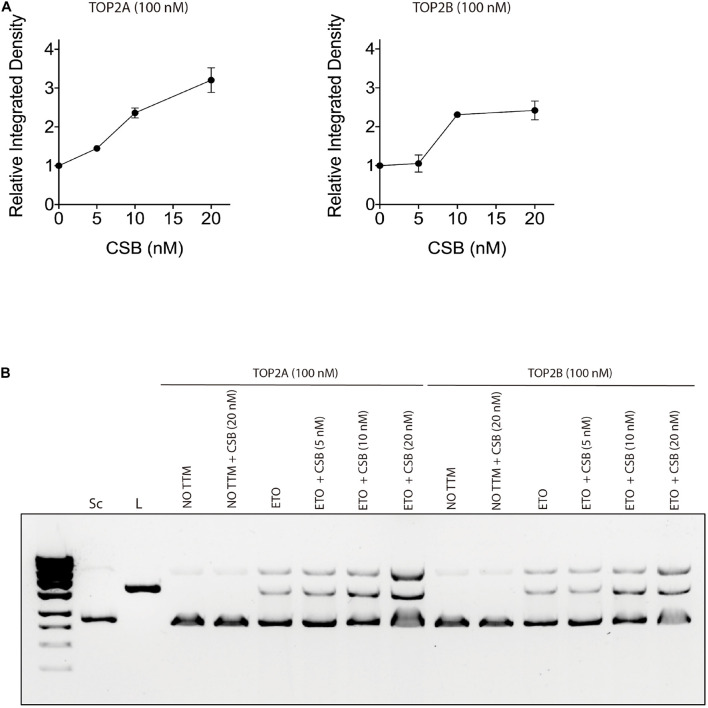
Cockayne Syndrome B stimulates TOP2A and TOP2B DNA cleavage in the presence of a TOP2 inhibitor treatment. **(A)** *in vitro* TOP2 cleavage assay quantification in the presence of CSB. **(B)** *in vitro* TOP2 cleavage assay representative gel. Sc, supercoiled DNA; L, linear DNA; NO TTM, no treatment; ETO, Etoposide.

In order to verify if CSB stimulates TOP2 function, we performed an *in vitro* cleavage assay with both proteins in the presence or not of the TOP2 inhibitor ETO. Our results show that CSB stimulates DNA cleavage by both TOP2 isoforms (alpha and beta) *in vitro* (Figure 5). This effect seems to happen since the addition of lower CSB concentrations (5 nM), but mainly at 10 nM for both isoforms, remaining still active in stimulating DNA cleavage at CSB 20 nM for TOP2A, as it is better observed by the quantifications of the image from Figure 5B, presented in Figure 5A. Although there is a trend that CSB presence stimulates more TOP2A than TOP2B, it was not statistically different. We can also see that the presence of CSB without the damage generated by ETO does not affects DNA cleavage. This indicates again that this interaction tends to occur in the face of any TOP2 interaction that might end in DNA damage, analyzed here by the trapping of TOP2 in the DNA caused by the ETO treatment.

## Discussion

### R-Loops and Topoisomerase 2 Inhibition/Knockdown

Nucleotide Excision Repair (NER) is known to repair adducts and bulky lesions in the DNA, that can occur at different parts of the genome. When these lesions are at transcribed active genes it causes RNA Pol arrest, and in response to this event CSB is recruited to start the signaling to other NER proteins that will remove the DNA containing the lesion. However, recent evidences have shown different roles for CSB (Sollier et al., 2014; Batenburg et al., 2015, 2017; Wei et al., 2015, 2016; Teng et al., 2018).

Sollier et al. (2014) have shown an involvement of CSB and other NER factors to remove R-loops that can be generated by different mechanisms, including inhibition of TOP1. Based on that and our previous interest in TOP2 inhibitors mechanisms and NER involvement to solve the induced lesions, we wondered if we could identify R-loops without an active TOP2 enzyme. Therefore, we investigated R-loops levels through two different methodologies, one using TOP2 inhibition with ETO, besides their knockdowns (through DRIP-qPCR), and the other with TOP2 knockdowns but inducing a different type of damage (through DART assay).

In our analysis, we could conclude that TOP2 presence is important to avoid R-loops formation/accumulation. However, its inhibition does not seem to change much as its knockdown. This makes sense considering that TOP2 is an important enzyme to keep DNA topology. Hence, its complete absence makes more difference than its inhibition that creates other lesions besides the complexes in the DNA. Although it was not evaluated in our study, it is still worth mentioning the role of Tdp2 protein, an important endonuclease that can remove Top2ccs (Nitiss and Nitiss, 2013; Pommier et al., 2014). Therefore, an interesting approach would be analyzing R-loops formation in response to TOP2 inhibition in the absence of Tdp2. This approach could show if the Top2ccs generated are removed by the endonuclease Tdp2 and how it influences the generation of R-loops in the presence or not of CSB.

Marinello et al. (2016), have seen increased R-loops formation in response to Campthotecin, a TOP1 inhibitor, after 2 and 10 min, but this was completely lost after 4 h of treatment, at different loci, including RPL13A, which is one locus also analyzed in our study. Based on that they affirm that TOP1 inhibition by Campthotecin can stabilize antisense and sense R-loops at active divergent promoters, but only for a short time (Marinello et al., 2016). We also analyzed R-loops levels at short and even longer-term ETO treatments through DRIP-qPCR. However, the difference in time does not seem to change the results in our case. It would be interesting to evaluate it in an even shorter time, such as 1 or 2 min after drug exposure. On the other side, it is known that R-loops are dynamic structures that are continuously formed and resolved and that the retention of nascent transcripts at their site of transcription is also a dynamic feature of the mammalian chromatin (Chédin, 2016).

It is known that negative supercoiling in the DNA (DNA under-winding) stabilizes R-loops, while positive supercoiling (DNA over-winding) tends to resolve them (Belotserkovskii et al., 2018). This could explain why different studies have observed R-loops induction after inhibiting TOP1 and we have not seen the same after TOP2 inhibition. However, this does not explain our findings in TOP2 knockdowns accumulating more R-loops in 4 of the 5 analyzed locus and at the specific TA-KR locus of the DART assay, which is transcriptionally activated.

We observed in DART assay results that siTOP2B cells presented more R-loops measured by S9.6 intensity in the KR foci area at the transcription activated locus. Although, in DRIP-qPCR, it was siTOP2A that showed higher levels of R-loops. This can be explained by the fact that TOP2B is more related to transcription, while TOP2A is usually related to replication. When we look at the data in tetR locus at DART assay, where transcription is not activated, we do see siTOP2B cells presenting a similar level of S9.6 intensity to the control. However, siTOP2A cells still present a slightly higher signal, which suggests that the absence of TOP2A really makes a difference in terms of R-loops formation/resolution, independently of transcription-activated or not. We did not evaluate a condition with transcription inhibition.

### Cockayne Syndrome B Stimulates Topoisomerase 2 DNA Cleavage Preventing Genome Instability

We have previously shown that CSB deficient cells accumulate more Top2ccs in response to MXT than XPC deficient or NER proficient cells (Rocha et al., 2016a). We investigated in this work if there is a physical interaction of CSB and TOP2 that could explain the difficulty in resolving these complexes generated by TOP2 inhibition with DOX, MXT, and ETO. ETO is known to be more specific in creating Top2ccs as this is its primary toxic mechanism, while DOX and MXT can create other lesions besides these complexes in the DNA (Parker et al., 1999; Bromberg et al., 2002; Minotti et al., 2004; Baldwin and Osheroff, 2005; Swift et al., 2006).

**FIGURE 6:**
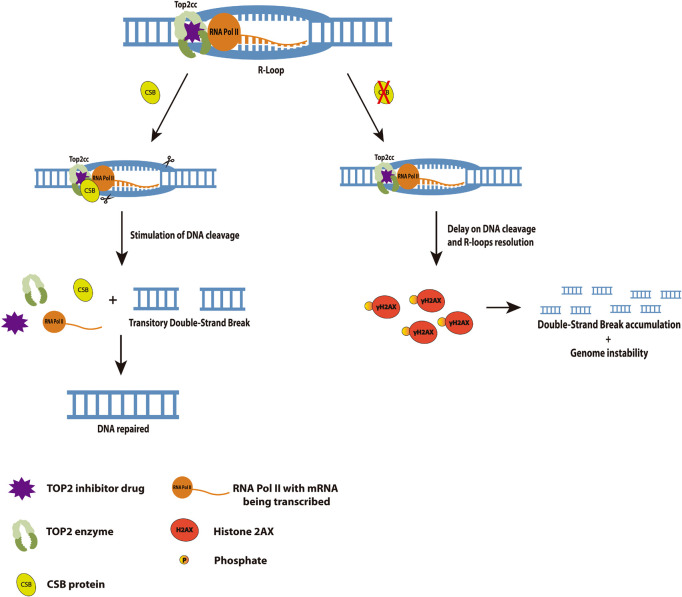
Cockayne Syndrome B interaction with TOP2 at a transcription arrest provoked by a TOP2 inhibition favors DNA cleavage, R-loops resolution and avoids genome instability. We propose here in this model that in the presence of CSB, the TOP2 inhibition generated lesions would be easily removed by TOP2 cleavage stimulated by CSB. These lesions could be R-loops formed by the hybridization of mRNA at the transcription bubble, since it is known RNA Pol II arrests when it encounters a lesion at the transcription active region. It is also known that this arrest recruits CSB, so in its absence, cells would accumulate R-loops for longer and consequently generates long-term DSBs (measured in our study by γH2AX), leading to genome instability.

We did find the interaction through co-IP of CSB and TOP2 after TOP2 inhibition and we also showed that CSB stimulates TOP2 DNA cleavage *in vitro*. The TOP2 inhibition creates Top2ccs in the DNA which is known that can block transcription, promoting then RNA PolII arrest. Taking these facts in consideration, we speculate at first that this arrest could favor R-loops formation and recruit CSB to this system. CSB presence could be essential to help in the removal of these R-loops by recruiting then other factors such as endonucleases that could relieve the super torsions, that result from the TOP2 trapped in the DNA, and consequently remove the R-loops. Considering that and our findings at the *in vitro* TOP2 cleavage assay, we could raise the hypothesis presented at our final model in Figure 6. In the presence of CSB, Top2ccs can be more easily removed after a Top2 inhibition once CSB stimulates the DNA cleavage by TOP2. This release of the Top2cc from the DNA would not favor for the R-loops accumulation, and cells might have some transitory DSBs during the process. However, when CSB is absent, the lack of stimulation for TOP2 to cleave the DNA might impact the accumulation of Top2ccs and R-loops and as a consequence DSBs generation that last longer and could end up in genome instability.

More studies are needed to elucidate better CSB or NER involvement in lesions mediated by TOP2 inhibition. Although we could not prove the formation of R-loops in TOP2 inhibition treatments and the participation of CSB in this process, we did show a functional interaction of CSB and TOP2. This interaction might be important to release TOP2 from the DNA when trapped due to an inhibition, for example. We also showed in this work the importance of TOP2 presence in preventing R-loops accumulation.

## Data Availability Statement

The raw data supporting the conclusions of this article will be made available by the authors, without undue reservation.

## Author Contributions

FB was responsible for main idea conceptualization, performing all methodologies except for the TOP2 cleavage assay, data analysis, making figures and writing-original draft preparation. SM was responsible for conceptualization and support at the DRIP-qPCR methodology and for reviewing the manuscript. YS and YP were responsible for performing TOP2 cleavage assay. JYM and JS were responsible for main idea conceptualization, supervision, writing, reviewing, and editing. All authors contributed to the article and approved the submitted version.

## Conflict of Interest

The authors declare that the research was conducted in the absence of any commercial or financial relationships that could be construed as a potential conflict of interest.

## Publisher’s Note

All claims expressed in this article are solely those of the authors and do not necessarily represent those of their affiliated organizations, or those of the publisher, the editors and the reviewers. Any product that may be evaluated in this article, or claim that may be made by its manufacturer, is not guaranteed or endorsed by the publisher.
