# Supplementary material for: Functions of the CSB Protein at Topoisomerase 2 Inhibitors-Induced DNA Lesions
Source: Front Cell Dev Biol. 2021 Oct 22;9:727836. doi: 10.3389/fcell.2021.727836 (PMC8569893; doi:10.3389/fcell.2021.727836)
Supplement: Supplementary file 1 [file Data_Sheet_1.docx]

**Supplementary Material**

## Supplementary Table

**Supplementary Table 1**. **Primers used for DRIP-qPCR**

| Loci | Forward | Reverse |
| --- | --- | --- |
| RPL13A | AGGTGCCTTGCTCACAGAGT | GGTTGCATTGCCCTCATTAC |
| NEAT1 | GGTGGCAGTGCTCCTTTTGG | CAGGCAAGCAACACCGATCC |
| HIST1H1E | CGCTGGCTATGACGTGGAGA | GCCTTCTTGGGCTTCTTCGC |
| HIST12BG | TGTGACCAAGGCGCAGAAGA | GAGCGCTTGTTGTAGTGGGC |
| EGR1 | TTCGGATTCCCGCAGTGT | TCACTTTCCCCCCTTTATCCA |

## Supplementary Figures


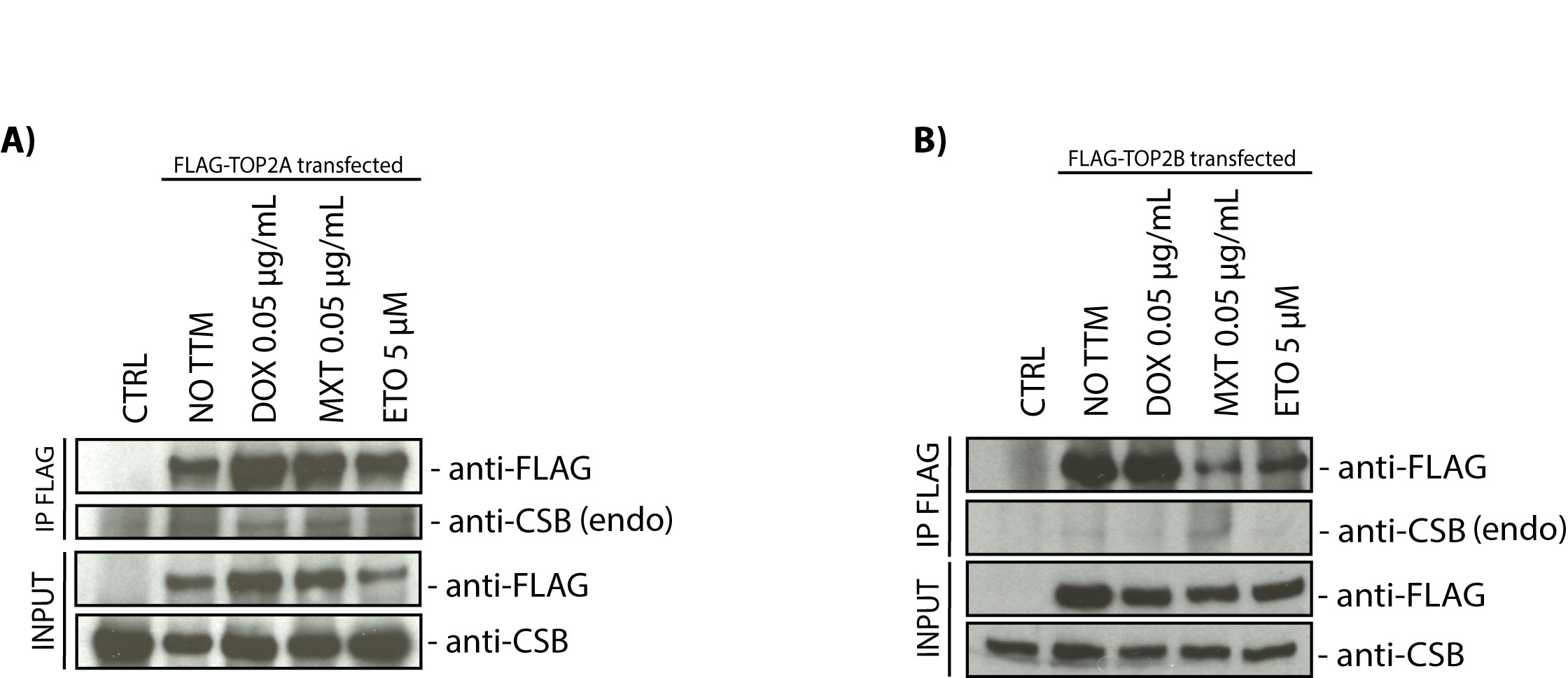


**Supplementary Figure 1**. **CSB physically interacts with TOP2A and TOP2B.** HEK-293T cells were transfected with pTREX vector to overexpress TOP2A (A) and TOP2B (B) FLAG-tagged proteins, and treated for 2 h with DOX, MXT and ETO. IP FLAG was performed after the treatments. CTRL cells were not transfected with pTREX. NO TTM: cells were transfected with pTREX but no treatment was applied.


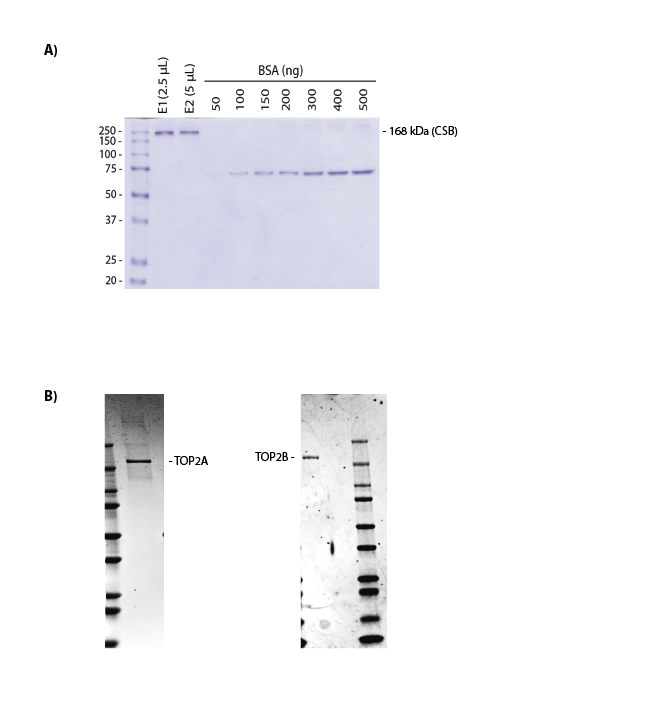


**Supplementary Figure 2**. CSB, TOP2A and TOP2B proteins purified. (A) SDS-PAGE gel of the full-length CSB protein purified by GST-His tagged method. E1 = elution 1 from purified CSB, E2 = elution 2 from purified CSB. (B) SDS-PAGE gel of purified TOP2A and TOP2B proteins.
